# Prevalence of dyslipidemia and hypercholesterolemia awareness: results from the Lookup 7+ online project

**DOI:** 10.1093/eurpub/ckab224

**Published:** 2022-01-29

**Authors:** Anna Maria Martone, Francesco Landi, Luca Petricca, Annamaria Paglionico, Rosa Liperoti, Maria Camilla Cipriani, Francesca Ciciarello, Sara Rocchi, Riccardo Calvani, Anna Picca, Emanuele Marzetti, Luca Santoro

**Affiliations:** 1 Fondazione Policlinico Universitario A. Gemelli IRCCS, Roma, Italy; 2 Università Cattolica del Sacro Cuore, Roma, Italy

## Abstract

**Background:**

Cardiovascular disease still represents the leading cause of death worldwide. Management of risk factors remains crucial; despite this, hypercholesterolemia, which is one of the most important modifiable cardiovascular risk factor, is still high prevalent in general population. The aim of this study is to determine the prevalence of dyslipidemia and hypercholesterolemia awareness in a very large population.

**Methods:**

More than 65 000 users completed the online, self-administered survey. It was structured like a ‘journey’ where each stage corresponded to a cardiovascular risk factor: blood pressure, body mass index, cholesterol, diet, physical exercise, smoke and blood sugar. At the end, the user received a final evaluation of his health status.

**Results:**

The mean age was 52.5 years (SD 13.9, range 18–98), with 35 402 (53.7%) men. About 56% of all participants believed to have normal cholesterol values, when only 40% of them really showed values <200 mg/dl. Only about 30% of all participants self-predicted to have abnormal cholesterol values whereas we found high cholesterol levels in about 60% of people.

**Conclusions:**

Dyslipidemia is very prevalent and half of the people with high cholesterol is not aware of having high values.

## Introduction

Cardiovascular disease (CVD) still represents the leading cause of death worldwide;^1^ in view of this, several prevention strategies have been implemented in the last decades with encouraging results, although mortality and morbidity rates due to CVD should still be improved.^2–5^ In fact, CVD, primarily atherosclerotic CVD, is responsible for four millions of deaths in Europe every year.^6^^,^^7^

The pivot point in primary and secondary CVD prevention remains the management of risk factors; among these last, adequate blood cholesterol levels achievement is strongly recommended, this goal being particularly significant when older subjects are involved.^8^^,^^9^

It is well known that lipid deposition, together with endothelial dysfunction, represents the first event that leads to initiation, growth and progression of atherosclerotic plaques in the artery wall.^10^^,^^11^

The 2019 ESC/EAS Guidelines^6^ underlines how low-density lipoproteins (LDL) play a causal role in atherosclerosis and how the duration of exposure to high levels of LDL cholesterol determines the extent of cardiovascular (CV) risk. These last guidelines contain a change of perspective in favor of an early cholesterol-lowering treatment, emphasizing the role of the absolute reduction of LDL cholesterol in determining the clinical benefit. ‘The lower, the better’ is the key concept considering that the amount of CV risk reduction is proportional to the amount of LDL cholesterol lowering, regardless of the basal concentration.^6^

In this respect, the document proposes a more aggressive therapeutic approach in order to determine a reduction of starting levels of LDL cholesterol by at least 50% in people at high or very high risk wherever patients with atherosclerotic CVD, diabetes with target organ damage, familial hypercholesterolemia and severe chronic kidney disease are all re-classified as high—very high risk.^6^^,^^12^

Despite these considerations, recent evidences show that awareness about importance of lipid profile is still low, especially in general health population.^13^^,^^14^

The aims of this study were to investigate the prevalence of hypercholesterolemia and to explore awareness of cholesterol levels through an online, self-administered survey in an unselected large sample of community-dwellers under the wider context of Longevity check-up 7+ (Lookup 7+) project.

## Methods

The Lookup 7+ project is an ongoing initiative developed by the Department of Geriatrics of the Catholic University of the Sacred Heart (Rome, Italy). The project started on 1 June 2015 and was designed to promote the adoption of healthier lifestyles by raising awareness in the general population on major lifestyle behaviors and risk factors for chronic diseases.^13^^,^^15^ Part of this projects has been developed in collaboration with Danone company, which hosted in its web site the web-survey (http://alcuoredelproblema.danacol.it/progetto-al-cuore-del-problema) that was set up in April 2017. The initiative was advertised in newspapers, magazines and TV broadcasting. The study protocol was approved by the Catholic University Ethics Committee (protocol #: A.1220/CE/2011). The manuscript was prepared in compliance with the STrengthening the Reporting of OBservational studies in Epidemiology reporting guidelines for observational studies.

Every person who wanted, could complete the above-mentioned online survey structured with close-ended answers and like a ‘journey’ called ‘A journey to the heart of the problem’ to put specific attention to CV implications. As part of the Lookup 7+ initiative, 65 892 individuals were enrolled between 1 June 2015 and 30 June 2017. We exclude all the subjects (*n* = 4321) with missing data for the variables of interest.

Each stage of the journey corresponded to a CV risk factor. The stages were:

FACTOR A: Blood pressure,

FACTOR B: Body Mass Index (i.e. Body Mass Index),

FACTOR C: Cholesterol,

FACTOR D: Balanced Diet,

FACTOR E: Exercise,

FACTOR F: Smoke,

FACTOR G: Blood sugar.

The user could watch introductory videos and learn more about the topic thanks to textual content for each item but, above all, he could carry out a self-check of his health status. The possibility of a self-check of the own health status aimed to stimulate a real change in incorrect lifestyles and a control of some health parameters correlated with CV health and, more generally, with successful longevity. Inclusion criteria were being aged 18 or older, having a device with internet connectivity, giving the online consent.

When id="287" the user started this ‘journey’ the first step was represented by information about handling personal data. Then, the user had to declare his/her gender, age (minors were not allowed) and residence region. The web questionnaire proceeded with smoking habit. It was categorized as current or never/former smoker. Then, the user was asked to answer about his diet, in particular if he consumed <2/3–4/>5 portions of fruit and vegetables per day. The conceptual assumption was that healthy diet is defined as the consumption of at least three portions of fruit and/or vegetables per day. Accordingly, to the Italian Society of Nutrition, three or more portions of fruit and/or vegetables correspond to more than 400 g, which is the minimum amount recommended by the WHO. Furthermore, the user had to answer the weekly frequency (expressed from 0 to 7) of consumption of the following foods: meat, egg, yogurt, rice, sausages, milk, pasta, legumes, cheese, bread and other cereals. The user was then asked if he practiced physical exercise for more than 2 h a week and if yes, which type: walking less than an hour a day, low-impact sports (e.g. yoga, tai chi and pilates), walking (more than 1 h a day), cardio activity (e.g. fast walking, running and swimming) and muscle strengthening activities (e.g. weight lifting).

The subsequent question was about the blood pressure: ‘Do you have measured your blood pressure during the last month?’ If the answer was ‘yes’, the user had to declare its value otherwise he had to declare usual blood pressure values (if < or > 120/80 mmHg), possible antihypertensive therapy.

In the next step, the height in centimeter and the weight in kilogram had to be declared.

Finally, the responders were asked about cholesterol and glucose levels awareness and measurements. In particular, participants were asked two questions about their cholesterol awareness:^1^ ‘How do you think your cholesterol level is?’ with possible answers being: ‘High’, ‘Normal’ or ‘I do not know’; and^2^ ‘Did you measure cholesterol in the last year?’ with possible answers being ‘Yes’ or ‘No’. If the answer was ‘yes’ the user had to declare its value.

Similar questions were present for glucose assessment and levels.

At the end of the survey, the following findings were considered as ideal CV health metrics: never/former smoker, regular engagement in physical activity, body mass index (BMI) 18.5–24.9, healthy diet, untreated total blood cholesterol <200 mg/dl, absence of diabetes and untreated blood pressure <120/80 mmHg. One point was assigned to each ideal metric, while a score of 0 was attributed to non-ideal categories. The final total score allowed to place the user in three different ‘zones’: (i) red (Scores 0–2): high risk for his/her health so the user was asked to contact his/her doctor to change his lifestyle urgently; (ii) yellow (Scores 3–5): intermediate health risk; and (iii) green (Scores 6 and 7): the user had shown correct behaviors; therefore, he was encouraged to continue his/her lifestyle.

### Statistical analyses

Continuous variables are expressed as mean ± SD, while categorical variables are shown as frequencies by absolute value and percentages. Descriptive statistics were used to describe demographic and key clinical characteristics of the study population according to gender. Differences in proportions and means of covariates between genders were assessed using the Fisher’s exact test and *t*-test statistics, respectively.

The primary focus of the analytic plan was to explore the prevalence of high blood cholesterol across self-predicted cholesterol levels among individuals who had not checked their cholesterol in past year. Participants were grouped by blood cholesterol levels [<200mg/dl (normal); 200–240 mg/dl (moderate high); >240 mg/dl (high)].

Logistic regression analysis was used to assess the association between clinical and lifestyle characteristics and cholesterol awareness. Univariate and adjusted models were performed for self-predicted cholesterol levels and for cholesterol checks in the past year. Candidate variables to be included in the logistic regression models were selected on the basis of their plausibility as risk factors for poor cholesterol awareness. We first estimated a crude prevalence rate ratio at 95% CI and then controlled for age and gender. Finally, logistic regression analyses were computed including all the variables of interest (age, gender, smoking habit, healthy diet, physical activity, BMI, blood pressure and diabetes).

All analyses were performed using SPSS software (V.18.0, SPSS, Chicago, IL, USA).

## Results

Overall, 65 892 users completed the online, self-administered survey. The characteristics of the participants are reported in table 1. The mean age was 52.5 years (SD 13.9, range 18–98), with 35 402 (53.7%) men.

**Table 1:** General characteristics of study sample according to gender and age

Characteristics	Total sample (*n* = 65 892)	Men (*n* = 35 402)	Women (*n* = 30 490)	*P*-values	Age <65 years (*n* = 52 838)	Age >65 years (*n* = 13 054)	*P*-values
Age	52.5 ± 13.9	53.9 ± 13.6	50.9 ± 14.1	<0.001	48.1 ± 11.8	70.2 ± 4.7	<0.001
Smoking	14 580 (22)	7375 (21)	7205 (23)	<0.001	12 628 (24)	1952 (15)	<0.001
Physically active	24 113 (36)	14 779 (42)	9334 (31)	<0.001	19 550 (37)	4563 (35)	<0.001
Healthy diet	26 412 (40)	13 100 (37)	17 208 (43)	<0.001	20 259 (38)	6153 (47)	<0.001
BMI	25.6 ± 4.5	26.4 ± 3.9	24.8 ± 4.9	<0.001	25.5 ± 4.5	26.4 ± 4.1	<0.001
SBP (mmHg)	126 ± 14	129 ± 13	122 ± 15	<0.001	124 ± 14	131 ± 13	<0.001
DBP (mmHg)	77 ± 9	79 ± 8	75 ± 9	<0.001	77 ± 9	77 ± 8	<0.001
Blood glucose (mg/dl)	97.9 ± 23.1	101.5 ± 24.4	93.2 ± 20.3	<0.001	95.6 ± 22.5	104.0 ± 23.6	<0.001
Cholesterol screening in past year (No)	25 146 (38)	12 260 (35)	12 886 (42)	<0.001	23 322 (42)	2824 (22)	<0.001
Cholesterol-lowering drugs	9762 (15)	5949 (17)	3813 (12)	<0.001	5700 (11)	4062 (31)	<0.001
Total blood cholesterol (mg/dl)	203.9 ± 43.3	199.4 ± 41.4	209.8 ± 45.0	<0.001	206.4 ± 43.8	196.1 ± 41.0	<0.001
Cholesterol level categories (mg/dl)							
2003 < 200	16 128 (40)	10 256 (45)	5872 (34)	<0.001	11 294 (37)	4834 (48)	<0.001
200–240	16 119 (41)	8931 (39)	7188 (42)		12 261 (41)	3858 (39)	
>240	7745 (19)	3573 (16)	4172 (24)		6442 (22)	1303 (13)	
Self-predicted cholesterol level							
Normal	36 992 (56)	20 680 (58)	16 312 (53)	<0.001	28 967 (55)	8025 (61)	<0.001
High	19 562 (30)	10 003 (28)	9559 (32)		15 829 (30)	3733 (29)	
Do not know	9380 (14)	4749 (14)	4631 (15)		8068 (15)	1312 (10)	

Notes: Data are given as the number (percent) for smoking, physical activity, healthy diet, cholesterol level categories, cholesterol-lowering drugs, cholesterol screening and self-predicted cholesterol; for all the other variables, means and standard deviations are reported. BMI, body mass index; SBP, systolic blood pressure; DBP, diastolic blood pressure.

When analyzing traditional CV risk factors, women were more frequently smokers than men (23% vs. 21%, respectively; *P* < 0.001), while men had mean blood glucose levels (101.5 ± 24.4 vs. 93.2 ± 20.3, respectively; *P* < 0.001), BMI values (26.4 ± 3.9 vs. 24.8 ± 4.9; *P* < 0.001) and systolic (129 ± 13 vs. 122 ± 15; *P* < 0.001) and diastolic (79 ± 8 vs. 75 ± 9; *P* < 0.001) blood pressure higher respect to women. Moreover, men were more frequently physically active than women (42% vs. 31%, respectively, *P* < 0.001), while women were more likely to follow a healthy diet as compared with men (43% vs. 37%, respectively, *P* < 0.001) (table 1). As for age, younger people had a higher percentage of smokers, while they showed lower BMI, systolic blood pressure and lower blood glucose values than older subjects (table 1).

The mean cholesterol level was higher in women than men (210 mg/dl vs. 199 mg/dl, respectively; *P* < 0.001). Condition of normal cholesterol levels (i.e. <200 mg/dl) was significantly more represented in men respect to women (45% vs. 34%, respectively; *P* < 0.001) (table 1). Younger people showed higher cholesterol values in comparison with older subjects (206 mg/dl vs. 196 mg/dl, respectively; *P* < 0.001). This finding is at least partly explained by the higher percentage of older subjects taking cholesterol-lowering drugs than younger subjects (31% vs. 11%, respectively; *P* < 0.001) (table 1).

**Figure 1:**
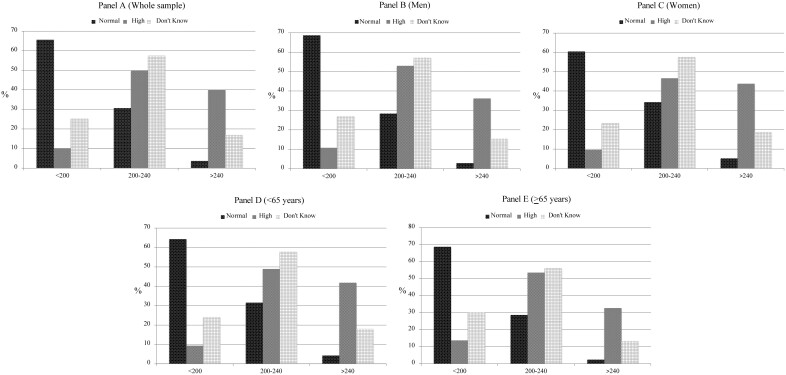
Total blood cholesterol according to self-predicted cholesterol levels in the whole sample (A), men (B), women (C), <65 years (D) and >65 years (E)

Interestingly, when considering self-predicted cholesterol levels, 56% of all participants believed to have normal cholesterol values, when only 40% of them really showed values <200 mg/dl (table 1). At the same time, more than 30% of all participants who thought to have normal cholesterol levels, actually showed values >200 mg/dl; on the other hand, about 10% of all participants showed normal cholesterol levels even if they thought to have abnormal values (figure 1).

With respect to gender differences about self-predicted cholesterol levels, 58% of men thought to have normal values compared with 53% of women (*P* < 0.001); similarly, 55% of younger subjects thought to have normal values compared with 61% of older people (*P* < 0.001) (table 1).

Interestingly, more than one-third of study population did not carry out a cholesterol check in the previous year, this condition being more common in women than men (42% vs. 35%, respectively, *P* < 0.001) and in younger than in older subjects (42% vs. 22%, respectively, *P* < 0.001) (table 1).

Table 2 shows predictive factors for normal self-predicted cholesterol levels. In the adjusted model, normal self-predicted cholesterol levels showed a direct association for female gender (OR 1.52, 95% CI 1.44–1.60), absence of smoking habit (OR 1.15, 95% CI 1.08–1.23), healthy diet (OR 1.12, 95% CI 1.07–1.18), physical activity (OR 1.26, 95% CI 1.20–1.33) and normal blood pressure (OR 1.24, 95% CI 1.18–1.31).

**Table 2:** Predictive factors for normal self-predicted cholesterol level

Variable	Self-predicted cholesterol normal (*n* = 36 992)	Self-predicted cholesterol high/do not know (*n* = 28 942)	Univariate odds ratio (95% CI)	**Adjusted odds ratio (95% CI)** ^a^
Age, years				
<65	28 947	23 878	1.0 (referent)	1.0 (referent)
>65	8015	5039	0.76 (0.73–0.79)	0.66 (0.63–0.70)
Gender				
Male	20 680	14 752	1.0 (referent)	1.0 (referent)
Female	16 312	14 190	1.21 (1.18–1.25)	1.52 (1.44–1.60)
Smoking habit				
Yes	8269	6299	1.0 (referent)	1.0 (referent)
No	28 723	22 643	1.03 (0.99–1.07)	1.15 (1.08–1.23)
Healthy diet				
No	21 271	18 250	1.0 (referent)	1.0 (referent)
Yes	15 720	10 692	1.26 (1.22–1.30)	1.12 (1.07–1.18)
Physically active				
No	22 120	19 073	1.0 (referent)	1.0 (referent)
Yes	14 872	9239	1.43 (1.38–1.48)	1.26 (1.20–1.33)
BMI (kg/m^2^)				
>25	16 795	14 600	1.0 (referent)	1.0 (referent)
≤25	17 653	12 383	1.23 (1.20–1.27)	1.05 (0.99–1.10)
Blood pressure				
High	17 527	14 399	1.0 (referent)	1.0 (referent)
Normal	14 612	10 031	1.19 (1.15–1.23)	1.24 (1.18–1.31)
Diabetes				
Yes	3381	2425	1.0 (referent)	1.0 (referent)
No	12 911	9910	0.93 (0.88–0.99)	0.96 (0.90–1.03)

a Adjusted simultaneously for all the variables listed.

Predictive factors for cholesterol screening in the previous year are shown in table 3. In the adjusted model, there was a direct association between cholesterol screening in the previous year with male gender (OR 1.37, 95% CI 1.27–1.49), age ≥65 years (OR 1.80, 95% CI 1.63–1.99), smoking habit (OR 1.38, 95% CI 1.26–1.51), high blood pressure (OR 1.21, 95% CI 1.11–1.31) and diabetes (OR 1.77, 95% CI 1.61–1.94).

**Table 3:** Predictive factors for cholesterol screening in previous year

Variable	Cholesterol checked (*n* = 40 807)	Cholesterol not checked (*n* = 25 146)	Univariate odds ratio (95% CI)	**Adjusted odds ratio** ^a^ **(95% CI)**
Age, years				
<65	30 556	22 322	1.0 (referent)	1.0 (referent)
>65	10 251	2824	3.16 (3.04–3.28)	1.80 (1.63–1.99)
Gender				
Female	17 623	12 886	1.0 (referent)	1.0 (referent)
Male	23 184	12 260	1.38 (1.34–1.42)	1.37 (1.27–1.49)
Current smoking				
No	33 238	18 135	1.0 (referent)	1.0 (referent)
Yes	7596	7011	1.69 (1.63–1.76)	1.38 (1.26–1.51)
Healthy diet				
No	22 778	16 762	1.0 (referent)	1.0 (referent)
Yes	18 029	8383	0.63 (0.61–0.65)	0.71 (0.65–0.77)
Physically active				
No	19 419	13 689	1.0 (referent)	1.0 (referent)
Yes	21 388	11 448	0.75 (0.73–0.78)	0.72 (0.66–0.78)
BMI (kg/m^2^)				
≤25	17 765	12 271	1.0 (referent)	1.0 (referent)
>25	20 345	11 050	1.27 (1.23–1.31)	1.01 (0.94–1.10)
Blood pressure				
Normal	15 786	8866	1.0 (referent)	1.0 (referent)
High	22 329	9599	1.30 (1.26–1.35)	1.21 (1.11–1.31)
Diabetes				
No	20 055	2766	1.0 (referent)	1.0 (referent)
Yes	4717	1089	1.67 (1.55–1.80)	1.77 (1.61–1.94)

a Adjusted simultaneously for all the variables listed.

## Discussion

In this online survey study, we investigated the prevalence of self-reported abnormal cholesterol levels and hypercholesterolemia awareness in an unselected, very large sample of community-dwellers. Overall, our study confirms that, despite the marked improvement of CV risk factors management in last decades, the presence of abnormal cholesterol levels remains a key point to be optimized; in our sample, we found high cholesterol blood levels in about 60% of participants, with mean cholesterol values being higher in women respect to men. These data are consistent with similar previous studies reporting low rate of normal cholesterol levels in general population^16^^,^^17^ and with the results obtained with direct measurements of cholesterol values from capillary blood samples of 6323 individuals enrolled in different Italian cities always in the context of Lookup 7+.^13^^,^^15^^,^^18^ These data confirm the need of further development of prevention strategies to better control one of the most important modifiable CV risk factor.^19^^,^^20^

Data about lipid profile awareness represent the key factor of our analysis and could explain, at least in part, the above-mentioned high rates of abnormal cholesterol levels. First of all, it is noteworthy that only about 30% of all participants self-predicted to have abnormal cholesterol values, whereas we found high cholesterol levels in about 60% of people: it means that half of the people with high cholesterol are not aware of having high values. These data are very impressive considering that lack of awareness about presence of a dyslipidemia condition could lead to a progression of subclinical atherosclerosis then evolving in CVD development.^10^^,^^21^ Besides, think to have a normal cholesterol profile leads screening to be less frequent, as showed in our study where, in agreement with previous surveys, about 40% of total sample do not control cholesterol values in the previous year.^8^^,^^16^^,^^22^ This behavior was particularly marked in subjects with healthy lifestyle habits, as healthy diet, physical activity and no smoking habit, while subjects with other pathological condition, as diabetes, high blood pressure and high BMI, showed greater care to regularly check their lipid profile.

As regards to gender differences, as already stated, our study showed that women, although younger compared to men, have higher cholesterol values. Interestingly, compared to men, women seem to be more conscious about the risk to have abnormal cholesterol levels; nonetheless, they check their lipid profile less regularly respect to men. This is likely, at least in part, to be related to the fact that hypercholesterolemia in postmenopausal women is considered an ‘inevitable corollary effect’ of the age. When considering age, in line with the Look Up 7+ cross-sectional survey, these results showed that with advancing age subjects are more inclined to check their cholesterol.^13^^,^^14^

Our findings about cholesterol awareness are similar to those from NHANES study^8^ and Look Up 7+ cross-sectional survey;^13^^,^^15^ there is still much to be done to improve CV health prevention strategies. In particular, we think that large scale awareness campaigns (especially addressed to the ‘critical’ age range between 40 and 60 years old), together with fast tracks for access to health checks, represent the goal to be strongly pursued. In this respect, an online, self-administered screening turned out to be particularly useful because it can be completed by anyone, from the comfort of their own home, and at any time. Not surprisingly, the strength of this study was the very large population sample.

The main limitation of our study is represented by the fact that data are self-reported, which could represent a recall bias. For example, we cannot assure that every respondent is aware of the portion size for consumption of fruits/vegetables and at the same time no information about quality is available. Regarding the self-reported measurement of blood pressure during the last month, there are many issues that may impact the quality of reporting data, such as recall bias of the actual value, tendency of under reporting the value and the actual use of antihypertensive therapy. Similarly, with respect to self-reporting of height and weight, there is usually the inclination to over reporting of height and under reporting of weight. With respect to the self- reporting the values of cholesterol, there is chance of under reporting the value, after going through their laboratory reports providing different cut-off values as normal and abnormal. Furthermore, we have no information about the laboratory and the time when the cholesterol has been assessed. However, overall our prevalence data are very similar to other survey, in which variables were measured directly and the use a web-survey allowed to involve a large sample of health individuals, which is the real strength of our analysis. The study sample declared the total cholesterol value and no information on LDL and high-density lipoprotein cholesterol was available. Nevertheless, total cholesterol is typically used for CV risk estimation in CVD risk prediction charts. In addition, we also have no information about the type of cholesterol-lowering medicines, e.g. whether the subjects were taking statins, ezetimibe or dietary supplements, such as plant phytosterols or red rice. Finally, the questionnaire was filled in by dwellers Caucasians and by people with technological skills therefore the results are not generalizable to other populations or to oldest old.

Even into the consideration of these limitations, our results suggest that people (women in particular) with a healthy lifestyle are more aware of their health and cholesterol levels, whereas those with alterations of more than one CV risk factor carry out checks more frequently. Probably the latter, due to their high-risk profile, have had a clinically relevant health problem linked to one or more of these alterations for which they are more monitored by their doctors.^23^

Lipid deposition is crucial for the onset and the progression of atherosclerosis, which leads to CVD development. The latest guidelines emphasize the role of the absolute reduction of cholesterol in the determining the clinical benefit. Awareness about lipid profile is still very low: half of the people with high cholesterol are not aware of having high values. Large scale awareness campaigns, together with fast tracks for access to health checks, represent an important goal to achieve.

## Funding

The Lookup 7+ online project was supported by Danone Italia. The study was also partly supported by intramural research grants from the Univerità Cattolica del Sacro Cuore (D3.2 2013 and D3.2 2015) to F.L. and by the non-profit research foundation ‘Centro Studi Achille e Linda Lorenzon’.

*Conflicts of interest*: None declared.

Key points

- Abnormal blood cholesterol is highly prevalent in Italian community-dwellers.
- Less than half of participants are aware of their cholesterol levels.
- The lack of awareness about presence of a dyslipidemia condition could lead to a progression of subclinical atherosclerosis.
- Large scale awareness campaigns represent an important goal to achieve for the CV prevention.
